# Supplementary material for: p300 KAT Regulates SOX10 Stability and Function in Human Melanoma
Source: Cancer Res Commun. 2024 Aug 1;4(8):1894–907. doi: 10.1158/2767-9764.CRC-24-0124 (PMC11293458; doi:10.1158/2767-9764.CRC-24-0124)
Supplement: Supplementary Figure S1 — This figure shows the correlation between EP300 and SOX10 gene copy numbers versus protein expression in melanoma cell lines as well as associations between PAK1 and GAB2 that do not correlate with EP300 or SOX10 gene copy numbers in melanoma cell lines. [file crc-24-0124_supplementary_figure_s1_suppsf1.pdf]

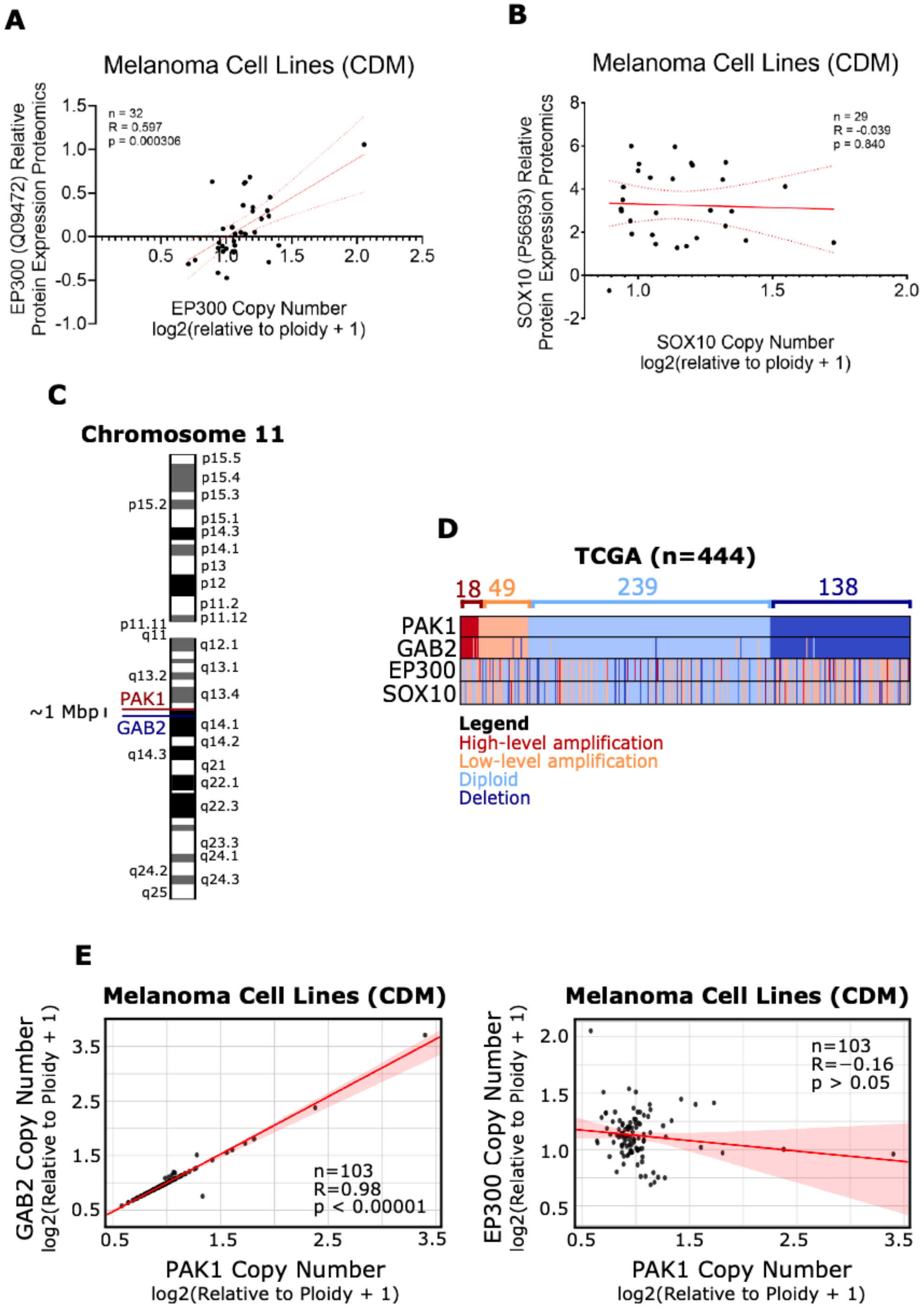
A
Melanoma Cell Lines (CDM)
n = 32
R = 0.597
p = 0.000306
EP300 (Q09472) Relative
Protein Expression Proteomics
EP300 Copy Number
log2(relative to ploidy + 1)
B
Melanoma Cell Lines (CDM)
n = 29
R = -0.039
p = 0.840
SOX10 (P56693) Relative
Protein Expression Proteomics
SOX10 Copy Number
log2(relative to ploidy + 1)
C
Chromosome 11
~1 Mbp
PAK1
GAB2
D
TCGA (n=444)
18
49
239
138
PAK1
GAB2
EP300
SOX10
Legend
High-level amplification
Low-level amplification
Diploid
Deletion
E
Melanoma Cell Lines (CDM)
n=103
R=0.98
p < 0.00001
GAB2 Copy Number
log2(Relative to Ploidy + 1)
PAK1 Copy Number
log2(Relative to Ploidy + 1)
Melanoma Cell Lines (CDM)
n=103
R=−0.16
p > 0.05
EP300 Copy Number
log2(Relative to Ploidy + 1)
PAK1 Copy Number
log2(Relative to Ploidy + 1)

Supplementary Figure 1: The copy numbers of PAK1 and GAB2 correlate with each other but not with EP300 or SOX10. (A) EP300 copy numbers positively correlate with EP300 protein levels in melanoma cell lines (data from Cancer Dependency Map [CDM]). **(B)** SOX10 copy numbers do not correlate with SOX10 protein levels in melanoma cell lines (data from Cancer Dependency Map [CDM]). **(C)** The PAK1 and GAB2 genes are located closely together on Chromosome 11. **(D)** PAK1 and GAB2 copy number levels are correlated in TCGA tumor samples, but they do not correlate with copy number levels of EP300 or SOX10. Copy number levels were defined by GISTIC2.0. **(E)** PAK1 and GAB2 copy numbers (left) are positively correlated in melanoma cell lines (data from Cancer Dependency Map [CDM]). EP300 and PAK1 copy numbers (right) are not correlated in melanoma cell lines (data from Cancer Dependency Map [CDM]).
