# Supplementary material for: p300 KAT Regulates SOX10 Stability and Function in Human Melanoma
Source: Cancer Res Commun. 2024 Aug 1;4(8):1894–907. doi: 10.1158/2767-9764.CRC-24-0124 (PMC11293458; doi:10.1158/2767-9764.CRC-24-0124)
Supplement: Supplementary Figure S2 — This figure shows the correlation of EP300 and SOX10 gene copy numbers in acral melanoma datasets. [file crc-24-0124_supplementary_figure_s2_suppsf2.pdf]

A

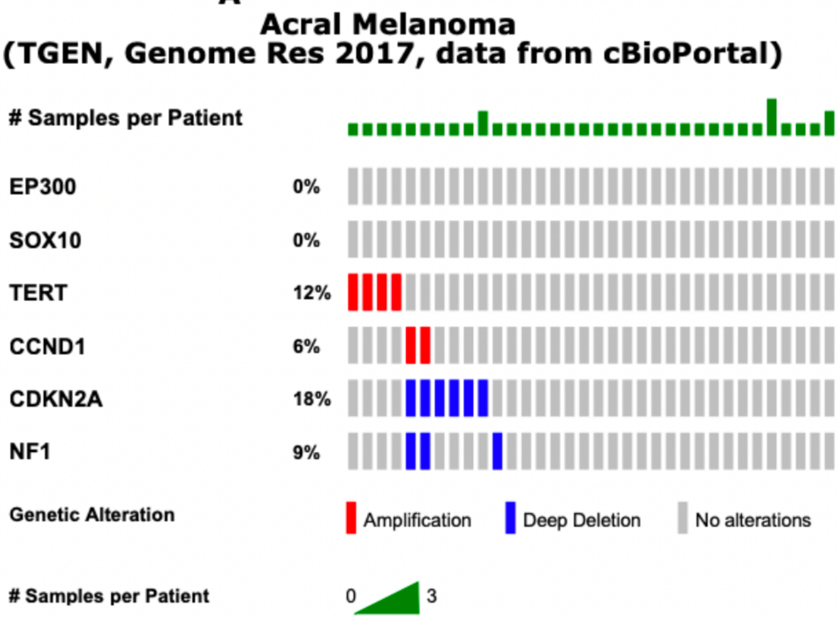
Acral Melanoma
(TGEN, Genome Res 2017, data from cBioPortal)
Samples per Patient
EP300 0%
SOX10 0%
TERT 12%
CCND1 6%
CDKN2A 18%
NF1 9%
Genetic Alteration
Amplification
Deep Deletion
No alterations
Samples per Patient
0
3

B

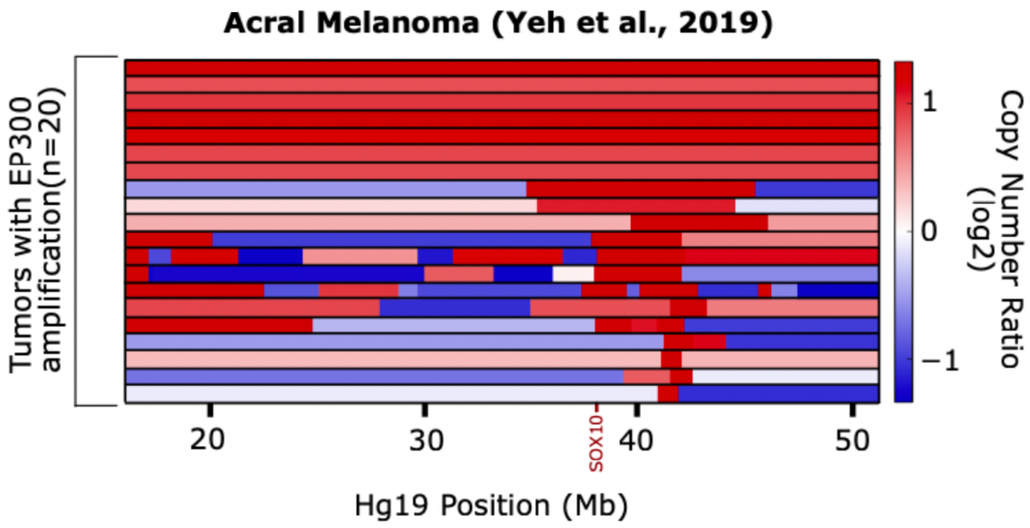
Acral Melanoma (Yeh et al., 2019)
Tumors with EP300 amplification(n=20)
1
0
−1
Copy Number Ratio (log2)
20
30
SOX10
40
50
Hg19 Position (Mb)

Supplementary Figure 2: EP300 amplification frequency is widely variable in acral melanoma datasets, but EP300/SOX10 co-amplifications are reproducible. (A) OncoPrint is shown for the TGEN Acral Melanoma Dataset (2017). Dataset is retrieved from cBioPortal. Copy number levels defined by GISTIC2.0. (B) EP300 and SOX10 copy numbers from acral melanoma patients in the Yeh et al. (2019) dataset.
