# Supplementary material for: p300 KAT Regulates SOX10 Stability and Function in Human Melanoma
Source: Cancer Res Commun. 2024 Aug 1;4(8):1894–907. doi: 10.1158/2767-9764.CRC-24-0124 (PMC11293458; doi:10.1158/2767-9764.CRC-24-0124)
Supplement: Supplementary Figure S3 — This figure illustrates the melanocytic cluster of genes located at human chromosome 22q13.1 and 22q13.2 [file crc-24-0124_supplementary_figure_s3_suppsf3.pdf]

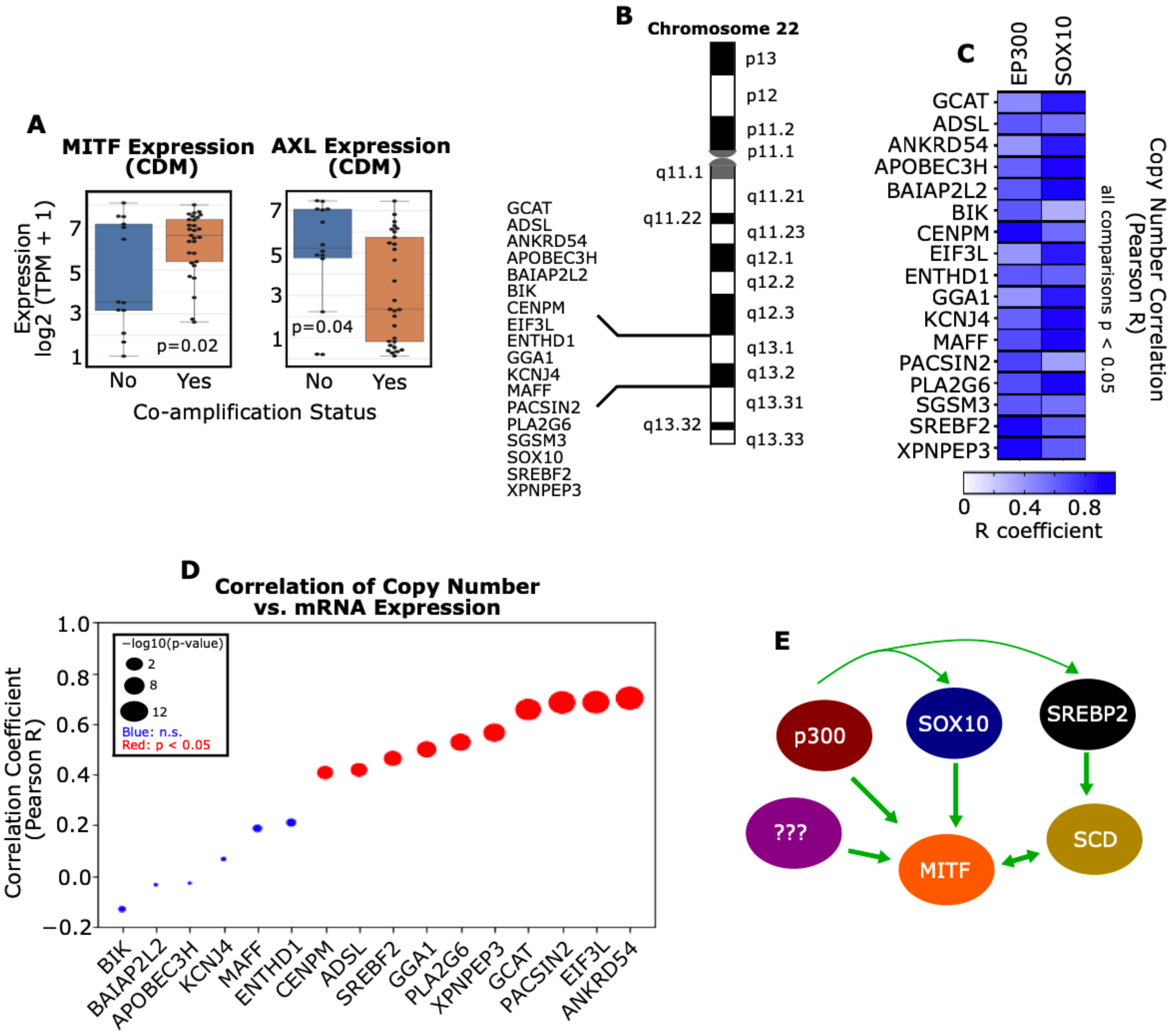
A
MITF Expression (CDM)
AXL Expression (CDM)
Expression log2 (TPM + 1)
p=0.02
p=0.04
No
Yes
Co-amplification Status
B
Chromosome 22
p13
p12
p11.2
p11.1
q11.1
q11.21
q11.22
q11.23
q12.1
q12.2
q12.3
q13.1
q13.2
q13.31
q13.32
q13.33
GCAT
ADSL
ANKRD54
APOBEC3H
BAIAP2L2
BIK
CENPM
EIF3L
ENTHD1
GGA1
KCNJ4
MAFF
PACSIN2
PLA2G6
SGSM3
SOX10
SREBF2
XPNPEP3
C
EP300
SOX10
Copy Number Correlation (Pearson R)
all comparisons p < 0.05
R coefficient
D
Correlation of Copy Number vs. mRNA Expression
Correlation Coefficient (Pearson R)
−log10(p-value)
Blue: n.s.
Red: p < 0.05
E
p300
SOX10
SREBP2
???
MITF
SCD

Supplementary Figure 3: Chromosome 22 q13.1 and q13.2 regions contain a melanocytic cluster of genes. **(A)** Cell lines with EP300/SOX10 co-amplifications have higher MITF and lower AXL expression than cell lines without co-amplifications (data from Cancer Dependency Map [CDM]). **(B)** q13.1 and q13.2 on Chromsome 22 contain 18 genes associated with the melanocytic phenotype in melanoma. **(C)** The copy number of the 18 melanocytic genes in q13.1 and q13.2 are positively correlated (data from Cancer Dependency Map [CDM]). **(D)** The correlation coefficient R is shown for the correlation of copy number versus mRNA expression for the 18 melanocytic genes (data from Cancer Dependency Map [CDM]). **(E)** At least EP300, SOX10 and SREBF2 genes are known regulators of MITF function in melanoma.
