# Supplementary material for: p300 KAT Regulates SOX10 Stability and Function in Human Melanoma
Source: Cancer Res Commun. 2024 Aug 1;4(8):1894–907. doi: 10.1158/2767-9764.CRC-24-0124 (PMC11293458; doi:10.1158/2767-9764.CRC-24-0124)
Supplement: Supplementary Figure S4 — This figure illustrates the effects of A-485 on SOX10 protein expression without effects on SOX10 gene expression. [file crc-24-0124_supplementary_figure_s4_suppsf4.pdf]

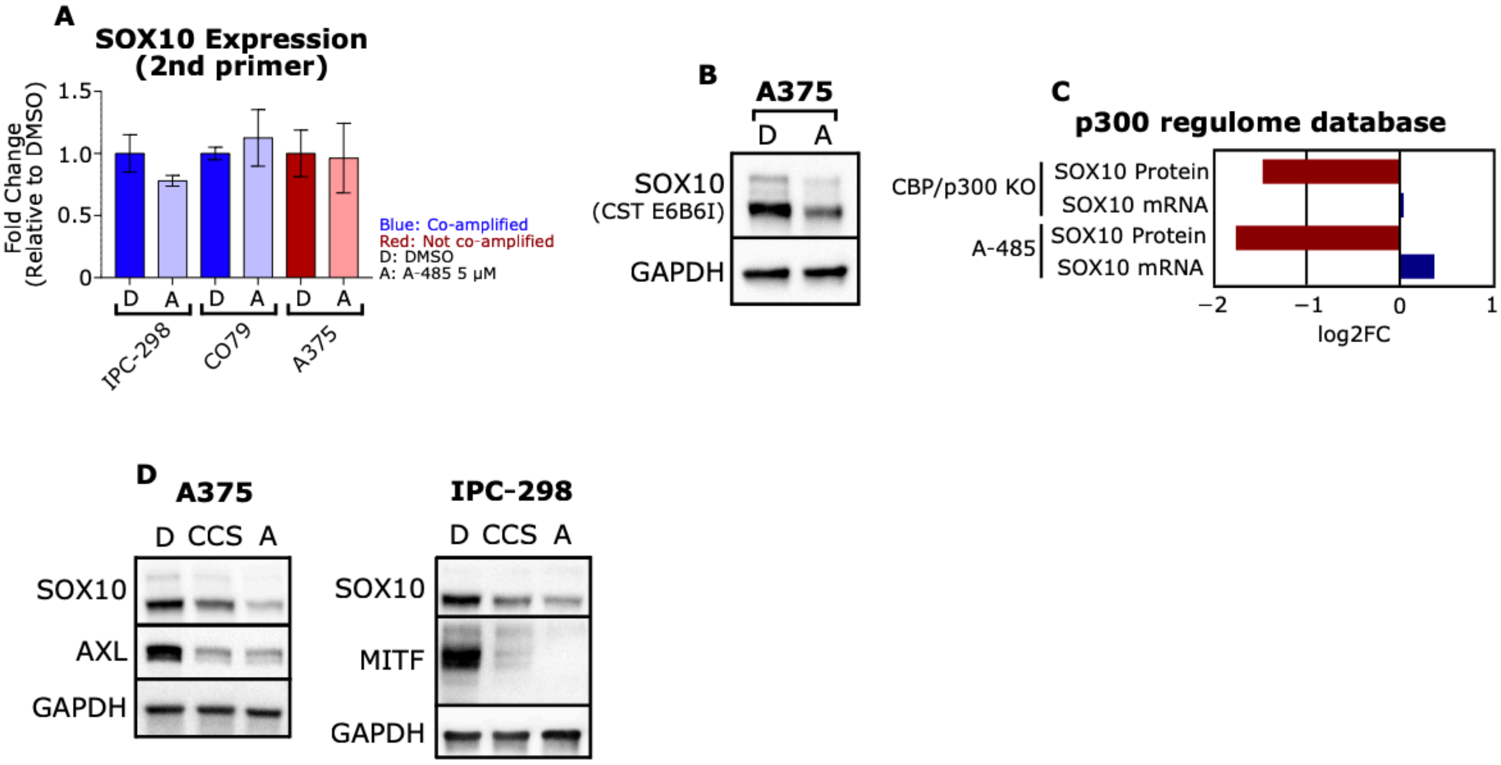
A
SOX10 Expression
(2nd primer)
Fold Change
(Relative to DMSO)
1.5
1.0
0.5
0
D
A
IPC-298
CO79
A375
Blue: Co-amplified
Red: Not co-amplified
D: DMSO
A: A-485 5 μM
B
A375
SOX10
(CST E6B6I)
GAPDH
C
p300 regulome database
CBP/p300 KO
A-485
SOX10 Protein
SOX10 mRNA
−2
−1
1
log2FC
D
CCS
AXL
IPC-298
MITF

Supplementary Figure 4: SOX10 is not transcriptionally downregulated by A-485, but is decreased at the protein level. (A) RT-qPCR validation of SOX10 mRNA expression following 5 μM A-485 treatment using a second qPCR primer. Data are represented as mean ± SEM. (B) Immunoblot validation of SOX10 protein levels following A-485 treatment using a second SOX10 antibody (CST 69661). D: DMSO, A: 5 μM A-485 **(C)** A-485 treatment and CBP/p300 knockout (KO) results in downregulation of SOX10 protein levels, but not decreased SOX10 mRNA expression in MEFs (data from p300 regulome database). **(D)** p300 bromodomain inhibitor CCS1477 (5 μM) downregulates SOX10 protein levels in melanoma cells and recapitulates the effects of A-485 on AXL and MITF in A375 and IPC-298 cells, respectively.
