# Supplementary material for: p300 KAT Regulates SOX10 Stability and Function in Human Melanoma
Source: Cancer Res Commun. 2024 Aug 1;4(8):1894–907. doi: 10.1158/2767-9764.CRC-24-0124 (PMC11293458; doi:10.1158/2767-9764.CRC-24-0124)
Supplement: Supplementary Figure S5 — This figure illustrates the correlation between p300 inhibition and subsequent downregulation of gene expression in SOX10-activated genes [file crc-24-0124_supplementary_figure_s5_suppsf5.pdf]

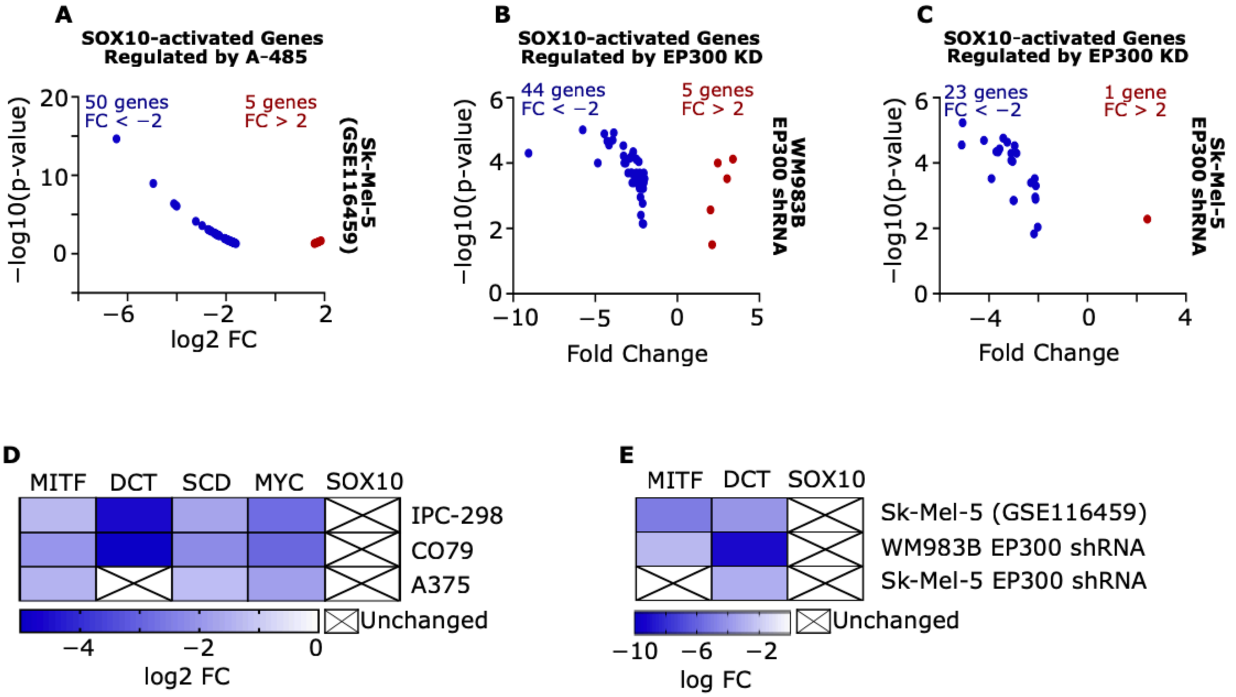
A
SOX10-activated Genes
Regulated by A-485
50 genes
FC < −2
5 genes
FC > 2
Sk-Mel-5
(GSE116459)
−log10(p-value)
20
10
0
−6
−2
2
log2 FC
B
SOX10-activated Genes
Regulated by EP300 KD
44 genes
FC < −2
5 genes
FC > 2
WM983B
EP300 shRNA
−log10(p-value)
6
4
2
0
−10
−5
0
5
Fold Change
C
SOX10-activated Genes
Regulated by EP300 KD
23 genes
FC < −2
1 gene
FC > 2
Sk-Mel-5
EP300 shRNA
−log10(p-value)
6
4
2
0
−4
0
4
Fold Change
D
MITF
DCT
SCD
MYC
SOX10
IPC-298
CO79
A375
−4
−2
0
log2 FC
Unchanged
E
MITF
DCT
SOX10
Sk-Mel-5 (GSE116459)
WM983B EP300 shRNA
Sk-Mel-5 EP300 shRNA
−10
−6
−2
log FC
Unchanged

Supplementary Figure 5: SOX10-activated genes are reproducibly downregulated by p300 inhibition across multiple datasets. (A) A volcano plot is shown for genes differentially expressed due to A-485 treatment in Sk-Mel-5 cells from GSE116459. Data analyzed by publicly available GEO2R. **(B)** Volcano plots are shown for genes differentially expressed due to EP300 knockdown in WM983B and Sk-Mel-5 cells (GSE128737). **(C)** SOX10-activated genes downregulated by A-485 in qPCR are reproducibly downregulated in our RNA-seq dataset (see Figure 2). Likewise, SOX10 is not downregulated by qPCR and is also unchanged in our RNA-seq dataset (see Figure 3). **(D)** SOX10 expression is unaltered by A-485 in GSE116459 or **(E)** EP300 KD in GSE1287373, but SOX10-activated genes are still reproducibly downregulated.
