# Supplementary material for: p300 KAT Regulates SOX10 Stability and Function in Human Melanoma
Source: Cancer Res Commun. 2024 Aug 1;4(8):1894–907. doi: 10.1158/2767-9764.CRC-24-0124 (PMC11293458; doi:10.1158/2767-9764.CRC-24-0124)
Supplement: Supplementary Figure S6 — This figure illustrates how A-485 leads to inhibition of expression of genes involved in melanoma cell invasion [file crc-24-0124_supplementary_figure_s6_suppsf6.pdf]

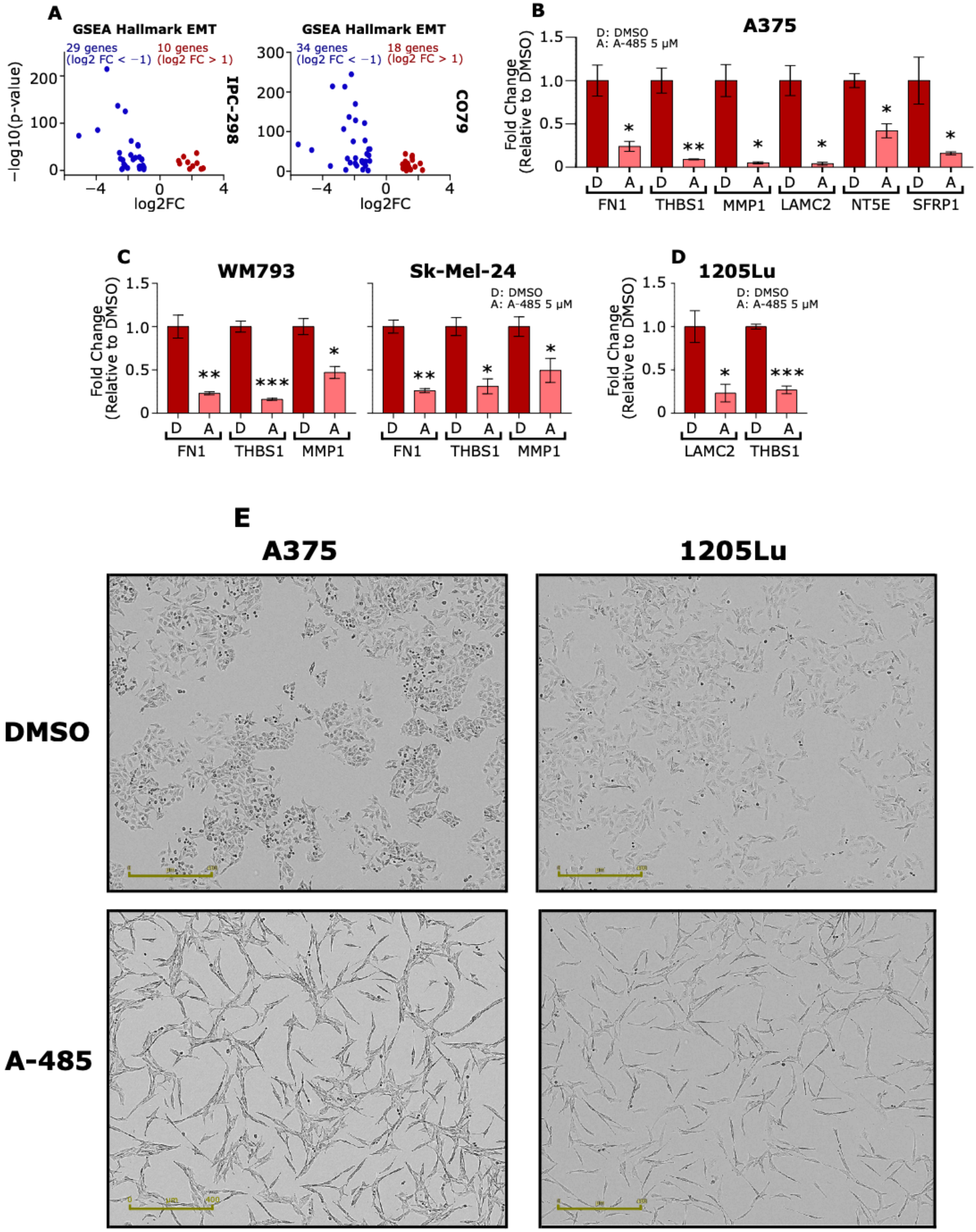
A
GSEA Hallmark EMT
29 genes (log2 FC < −1)
10 genes (log2 FC > 1)
IPC-298
GSEA Hallmark EMT
34 genes (log2 FC < −1)
18 genes (log2 FC > 1)
CO79
−log10(p-value)
log2FC
B
A375
D: DMSO
A: A-485 5 μM
Fold Change (Relative to DMSO)
FN1
THBS1
MMP1
LAMC2
NT5E
SFRP1
C
WM793
Sk-Mel-24
D: DMSO
A: A-485 5 μM
Fold Change (Relative to DMSO)
FN1
THBS1
MMP1
D
1205Lu
D: DMSO
A: A-485 5 μM
Fold Change (Relative to DMSO)
LAMC2
THBS1
E
A375
1205Lu
DMSO
A-485

Supplementary Figure 6: A-485 decreases expression of genes involved in invasion. (A) Volcano plots are shown for genes in the GSEA EMT Hallmark gene set that are differentially expressed due to A-485 treatment in MITF-high IPC-298 and CO79 cells. **(B)** RT-qPCR validation of SOX10-regulated EMT genes downregulated by A-485 in the RNA-seq in A375 cells. **(C)** RT-qPCR validation of SOX10-regulated EMT genes downregulated by A-485 in WM793 and Sk-Mel-24 cells. **(D)** RT-qPCR validation of SOX10-regulated EMT genes downregulated by A-485 in 1205Lu cells. **(E)** 5 μM A-485 alters the cell morphology of A375 and 1205Lu cells after long-term (12-day) treatment (larger images for comparison to Figure 6J). Data are represented as mean ± SEM. $*p < 0.05$; $**p < 0.005$; $***p < 0.0005$.
