# Supplementary material for: p300 KAT Regulates SOX10 Stability and Function in Human Melanoma
Source: Cancer Res Commun. 2024 Aug 1;4(8):1894–907. doi: 10.1158/2767-9764.CRC-24-0124 (PMC11293458; doi:10.1158/2767-9764.CRC-24-0124)
Supplement: Supplementary Figure S7 — This figure illustrates the acetylation sites in SOX10 and how SOX10 protein but not mRNA levels decrease with p300 inhibition. [file crc-24-0124_supplementary_figure_s7_suppsf7.pdf]

A

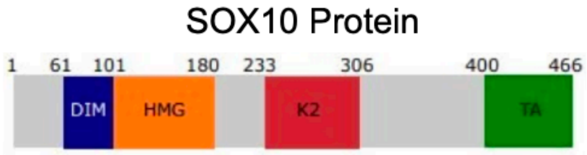

B

Lysine Residue	Acetylation (log2FC)	Mouse Sequence	Human Sequence
K140	−3.9	SKTLGKLWRLL	SKTLGKLWRLL

C

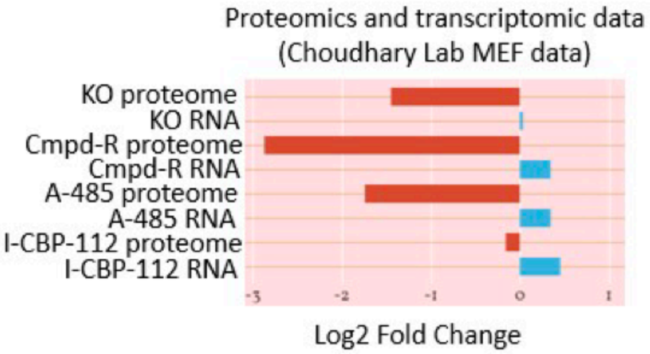

Supplementary Figure 7: SOX10 protein is acetylated and protein levels, but not mRNA, decrease with CBP/p300 KAT inhibition. (A) Structural modules of SOX10. Amino acids are based on the SOX10 protein sequence. (B) Change in acetylation of K140 SOX10 lysine residue in mouse embryonic fibroblasts (MEFs) treated with 3 uM A-485 for 16 h. (C) Changes in SOX10 protein or mRNA expression (Log2FC) in p300 KO MEFs (KO) or MEFs treated with 3 uM compound R (Cmpd-R), 3 uM A-485 (A-485), or 5 uM I-CBP-112 (I-CBP-112) for 16 h. Data mined from (Weinert et al., 2018; http://p300db.choudharylab.org).
