# Supplementary material for: p300 KAT Regulates SOX10 Stability and Function in Human Melanoma
Source: Cancer Res Commun. 2024 Aug 1;4(8):1894–907. doi: 10.1158/2767-9764.CRC-24-0124 (PMC11293458; doi:10.1158/2767-9764.CRC-24-0124)
Supplement: Supplementary Table S1 — EP300 amplification frequencies in several acral melanoma datasets. [file crc-24-0124_supplementary_table_s1_suppst1.pdf]

**Acral Datasets**	**% with EP300 amplification**
TGEN (2017)	0%<br>0/38 samples
Yeh et al. (2019)	16.4%<br>20/122 samples
Shi et al. (2022)	31.7%<br>19/60 samples

Supplementary Table 1. EP300 amplification frequencies in several acral melanoma datasets.
